# Understanding congested travel in urban areas

**DOI:** 10.1038/ncomms10793

**Published:** 2016-03-15

**Authors:** Serdar Çolak, Antonio Lima, Marta C. González

**Affiliations:** 1 Department of Civil and Environmental Engineering, MIT, Cambridge, Massachusetts 02139, USA; 2 School of Computer Science, University of Birmingham, Edgbaston B15 2TT, UK; 3 Engineering Systems Division, MIT, Cambridge, Massachusetts 02139, USA

## Abstract

Rapid urbanization and increasing demand for transportation burdens urban road infrastructures. The interplay of number of vehicles and available road capacity on their routes determines the level of congestion. Although approaches to modify demand and capacity exist, the possible limits of congestion alleviation by only modifying route choices have not been systematically studied. Here we couple the road networks of five diverse cities with the travel demand profiles in the morning peak hour obtained from billions of mobile phone traces to comprehensively analyse urban traffic. We present that a dimensionless ratio of the road supply to the travel demand explains the percentage of time lost in congestion. Finally, we examine congestion relief under a centralized routing scheme with varying levels of awareness of social good and quantify the benefits to show that moderate levels are enough to achieve significant collective travel time savings.

## Abstract (web summary)

Rapid urbanization burdens urban road infrastructures, but understanding the interplay of road infrastructure and travel patterns is a complex challenge. Here, authors use mobile phone traces during morning peak hours to evaluate the effect of a congestion relief approach under a centralized routing scheme.

## Graphical abstract

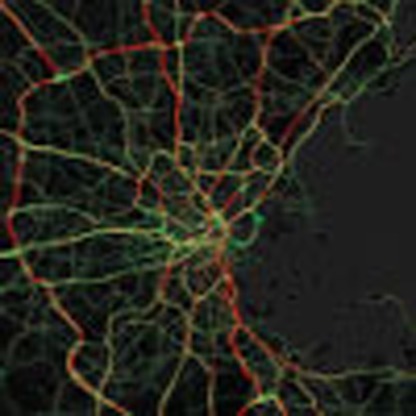

Cities have a long-standing history cultivating technological innovations that allow citizens to efficiently access goods and opportunities. However, the ease of access has been increasingly difficult to maintain under rapid urbanization^1^,^2^,^3^,^4^,^5^,^6^,^7^. As growing population densities create excessive demand for cities' infrastructure, the increasing penetration and advancement of technology generates massive amounts of multidimensional data that can be used to study and mitigate this demand. Specifically, the availability of mobile phone data has led researchers to quantify fundamental spatiotemporal patterns to better understand human mobility in urban areas^8^,^9^,^10^,^11^,^12^. With the continuous increase in the volume and accuracy of new data sources, new methods that process and distill mobile phone data are consistently refined, and traditional models of mobility such as the gravity-, radiation- or activity-based models are being updated in tandem^13^,^14^,^15^,^16^,^17^,^18^. In the context of travel demand estimation, previous efforts focused on developing models that combine household travel surveys with census and land-use information^19^,^20^. Despite the robust methodology and meticulous implementation of these models, the high costs associated with obtaining the infrequent and small data have proven to be the bottleneck. To supplement these approaches, traffic simulations and demand estimation models have begun incorporating big data sources into their forecasts, building portable data pipelines to create data-driven decision-making tools for policy makers^21^,^22^,^23^.

Understanding of the complex interplay of road infrastructure and travel patterns to model travel times and congestion in not a single city but many at once has been a particular challenge in this line of research^24^,^25^,^26^. Road networks, the circulatory system sustaining a city's accessibility and cultivating its economic prosperity^27^,^28^,^29^ are seized with congestion in most large metropolitan areas. In their 2013 report, TomTom, a leading GPS company, states that in cities such as Moscow, Istanbul, Rio de Janeiro, Mexico City and Beijing, people on average spend >75% extra time travelling due to traffic. The resulting loss of time, money and energy are borne by the city's citizens and travellers. Municipalities continually invest in road infrastructure construction and maintenance to increase supply, although controversies on whether more roads alleviate congestion persist^30^. Other efforts to reduce congestion aim to decrease driving demand by promoting alternative travel modes, high-occupancy driving lanes, carpooling, congestion pricing and, in extreme cases, road space rationing. Even with all these measures, congestion remains inherent and drivers are increasingly leveraging real-time information through GPS devices and online routing tools to move faster. With everyone having easy access to traffic information, drivers make decisions without coordination based on near-perfect information, resulting in suboptimal system configuration. This general trend of using raw real-time information in decision-making has significant implications, as it might be also used as a tool to guide drivers to make choices for the benefit of the city, thus creating a more optimal traffic configuration. The extent of the global inefficiency has been of great interest^31^,^32^,^33^,^34^ in many contexts, ranging from wireless networks to transportation^35^,^36^,^37^,^38^,^39^,^40^. Theoretical approaches to bring the system to optimality generally converge to marginal cost taxation, which essentially forms the basis of congestion pricing schemes today^41^,^42^. Despite the abundance of research on optimal flow configurations and their implications in the transportation, urban planning and economics literature, there is a shortage of works that use big data sources to understand the role of travel demand and actual travel times in metropolitan regions when comparing cities. This highlights a need to build a framework that can be replicated to systematically generate meaningful travel times to not only understand cities better but also test solutions to urban problems such as congestion or pollution.

In this work, we address this issue by coupling travel demand profiles and travel time estimates to analyse how efficiently people move across cities. We begin by modelling the supply by parsing publicly available OpenStreetMap data to obtain road networks. To model travel demand, we mine massive mobile phone data sets, also referred to as call detail records (CDRs)^43^. This procedure requires home and work location detection for millions of users, mining of their location shifts, and the proper sampling procedures to represent accurately the trip tables for the whole city (see Supplementary Notes 1–3). Using this information of the trip distribution within the city, we estimate morning peak vehicular volumes from origins to destinations and compare the inferred travel times based on demand with the estimates of an online map provider in the respective routes and hour of the day. We then explore the relationship between travel distance and travel time across many cities. We show that the time lost due to congestion in each city can be accounted by a dimensionless parameter Γ that measures the ratio between the vehicular travel demand and the road infrastructure supply for the city. To a lesser extent, the differences in congestion levels depend on the population density and the spatial distribution of population. Next, we calculate the detrimental effects of selfish routing by comparing obtained travel times to those that would be observed if the routes were selected to attain the social optimum. We then explore the bounds of the benefits of leveraging information technologies to influence route choices in ways that would help create a more optimal system configuration for vehicular travel. To do so, we implement a generalized selfish routing model that generates expected travel times for varying levels of consideration of overall social good, or *λ*. We analyse the system gains of socially aware driver behaviour, as well as exploring the distributions of benefits and losses at the individual level. We present our findings for ;five major cities around the world: Boston and San Francisco Bay Area in the United States, Rio de Janeiro in Brazil, and Lisbon and Porto in Portugal.

## Results

### Approach

We formalize the traffic problem by modelling route choice as follows: every driver *i* makes a choice of the route *p* to their destination. This choice depends on a personal utility 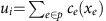, expressed as the sum of the costs *c* of every road segment *e* along the chosen route. For simplicity, we assume that the cost of a road segment for driver *i* is equal to the travel time, 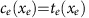, where *t*_*e*_(*x*_*e*_) represents the travel time *t* observed on road *e* for vehicle flow *x*_*e*_. We can then define the total cost incurred by all users as 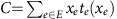. The flow configuration that results in the optimal cost is referred to as the socially optimal flows obtained by a typical minimum cost network flow programme^44^:


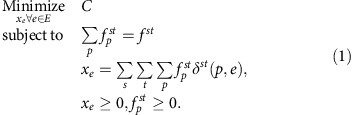


where *x*_*e*_ refers to the flow on road *e*, 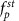 is the flow between the source *s* and target *t* on route *p*, and *δ*^*st*^(*p*, *e*)=1 when road *e* lies on route *p*.

As drivers make selfish choices, the system settles into a suboptimal state. Although driver *i* only experiences and considers his/her own travel time, the cost the whole system incurs also includes the marginal cost driver *i* imposes on all other drivers on the road segments he/she takes. The set of flows that occur when every driver minimizes their own travel time is referred to as the user equilibrium flows. Theoretically, in the resulting system state, no driver can benefit from deviating from their route. This idea, essentially describing a Nash equilibrium in roads, is captured in Wardrop's principles in transportation^36^: the journey times on all the used routes for an origin–destination (OD) pair are equal and are less than those that would be experienced by a single vehicle on any unused route. This routing game is solved through a potential function 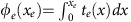 such that 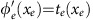
(ref. 45). The convex programme for the user equilibrium problem has been formulated^46^ as follows:


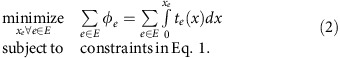


**Figure 1:**
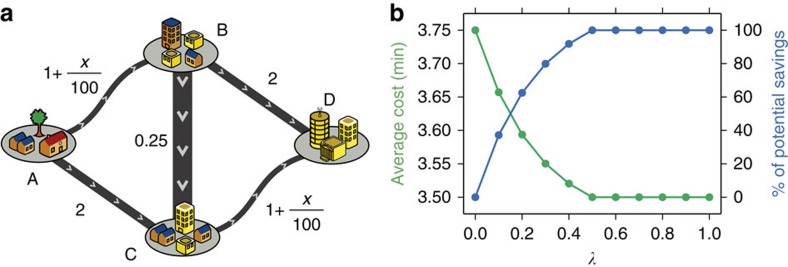
Illustration of routing equilibrium. (**a**) In this small network, 100 drivers are going from A to D. The road labels represent the costs of travel as a function of vehicle flows. User equilibrium allocates the flows between paths as *f*_ABD_=*f*_ACD_=25 and *f*_ABCD_=50, and the average travel time is 3.75 min for all drivers. Socially optimal flows decrease total travel time to 3.5 by *f*_ABD_=*f*_ACD_=50 and *f*_ABCD_=0, with road *BC* remaining unused. (**b**) Achieved percentage of potential savings for increasing values of social good weight *λ*: 10 and 20% social good weight results in 40 and 60% of potential savings, respectively.

Figure 1a depicts an example that captures solutions for equilibrium and optimal flows for a widely used toy network. For the demand of *d*_AD_=100, the user equilibrium flows allocate 50 drivers on path ABCD and 25 drivers on paths ABD and ACD each, resulting in a travel time from A to D of 3.75, regardless of the path chosen. The socially optimal configuration avoids allocating too much flow on the path ABCD, as its marginal cost is higher than those of paths ABD and ACD. By minimizing the marginal cost, path ABCD receives no flow and the average cost is minimized at 3.5.

To assess the benefits of different scenarios based on travel demand information, we make use of the formulation proposed in ref. 47. We reconfigure the utility function of a driver as a linear combination of the cost he/she will incur and the total marginal cost his/her choice imposes on everyone else:


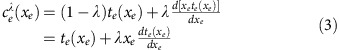


*λ* defines the weight towards social good; it is a parameter ranging between 0 and 1. A driver with *λ*=1 chooses routes with respect to the marginal costs, thus moving the system closer to the system optimum. Conversely, a user with *λ*=0 only considers the cost of his route and potentially moves the system away from optimality. The resulting convex programme for the socially aware routing problem is as follows:


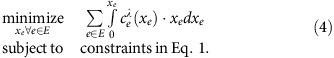


For the city depicted in Fig. 1a, the user equilibrium configuration results in an average cost of 3.75 min per driver versus 3.5 min the system optimum, meaning solely by adjusting routing behaviour to *λ*=1, a benefit of 0.25 min can be achieved per driver. Figure 1b shows that for *λ*=0.1, when the drivers begin valuing social good as well, the average cost drops to ∼3.65 and almost 40% of potential savings are realized. In fact, the social optimum is achieved at *λ*=0.5.

### Travel times

**Figure 2:**
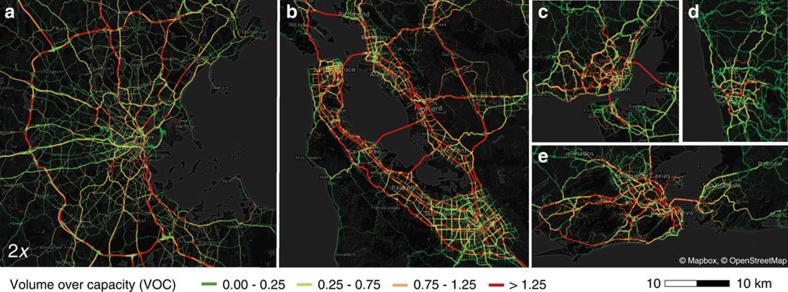
The maps of VOCs (volume over capacity) of the roads in the user equilibrium configuration. The depicted cities are (**a**) Boston, USA, (**b**) San Francisco Bay Area, USA, (**c**) Lisbon, Portugal, (**d**) Porto, Portugal, and (**e**) Rio de Janeiro, Brazil. Higher VOCs are generally observed in highways, as they provide faster means of travel. (Boston is 2*x* the distance scale.) Maps under © OpenStreetMap contributors BY-SA.

To understand the relationship between travel demand and driving travel times, we begin by comparing our five cities during estimated morning peak period traffic conditions. The areas of analysis are significantly diverse: Rio is very highly populated over its large extensions, whereas Porto's population density considerably decreases after *r*>20 km from the most dense location. Rio de Janeiro, the Bay Area and Lisbon extend across Guanabara Bay, the Bay and Tagus, respectively, and have many inhabitants commuting on few bridges (see Supplementary Fig. 3 and Supplementary Table 1 for more details). As a consequence of their differences, cities demonstrate varying traffic conditions, as shown in Fig. 2. The volume-over-capacity ratio (VOC) measures how successfully a road segment is able to cope with the assigned volume of vehicles, with high VOC values indicating more congestion. High VOCs are generally observed on highways, as they provide faster means of travel due to their wider roads, increased number of lanes and higher speed limits. In addition, bridges and roads that lie central in the network topology are typically congested due to a lack of alternative routes.

**Figure 3:**
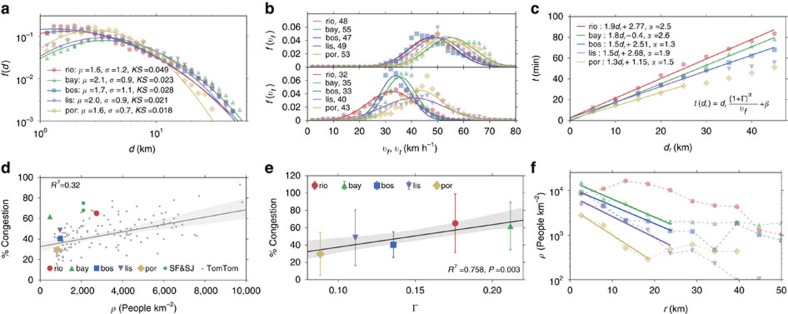
Comparisons of cities and their congested travel. (**a**) Distributions of commuting trip distances, *d*, in the morning peak period with parameters of the fitted lognormal distribution depicted in the legend (see Supplementary Fig. 7 and Supplementary Table 2 for more detail). (**b**) Distribution of trip free flow speeds, *v*_*f*_, and in traffic conditions, *v*_*t*_. (**c**) Commuting travel times versus route distances of commuters, *d*_*r*_. (**d**) Estimates of overall mean % of time lost in congestion versus population density *p* for TomTom Traffic Index estimates and our analysis. (**e**) Relationship of overall mean % congestion to the demand to supply ratio, Γ, for the five subject cities, with error bars specifying the s.d. (see Supplementary Fig. 8). (**f**) Average population density *ρ* as a function of distance from the most dense area in the region, *r*.

We begin by analysing the efficiency of urban mobility for the five regions to understand the mechanisms underlying observed travel times. The main determinant of congestion is travel demand, which is heavily tied to commuting trip distances during weekday peak travel times. In Fig. 3a, we demonstrate that the straight-line (Euclidean) commuting distances, *d*, follow a lognormal distribution, 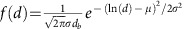 with means ranging from 5 to 8 km (*μ*=1.6–2.1) and s.d. ranging from 2 to 4 km (*σ*=0.7–1.2) (see Supplementary Fig. 7). It can be observed that majority of trips span relatively short distances and trips over 25 km are uncommon. However, what makes a city more traversable are the speeds at which drivers can span these distances. In Fig. 3b we investigate the effective speeds in both free and congested traffic conditions. It can be observed that cities exhibit similar free travel-speed distributions, normally distributed with *μ* fluctuating around 50 km h^−1^ with mean values reported in the legend. The differences in road network supply 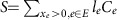 (km vehicles per hour), where *l*_*e*_ and *C*_*e*_ are the length (km) and the flow capacity (vehicles per hour) of a road segment *e*, explains the slight differences in free flow speeds, as seen in Table 1. These differences are significantly more apparent in speed distributions under real traffic conditions: the effective OD travel speeds in Rio, the Bay Area and Boston decay considerably compared with those in free traffic conditions, whereas the speeds in Porto and Lisbon change less. We explore further these two different responses given the demand profiles of each city.

To that end, we analyse the experienced travel times per distances travelled in Fig. 3c. We observe a strong yet very simple relationship that pronounces the differences between the subject cities: Rio de Janeiro is the slowest city and is followed next by the Bay Area, and Porto is the fastest. All cities exhibit a linear relationship, with the exception of long-distance trips in Porto and Lisbon where a different regime appears for longer distances. To explain this observation, we model travel times by city-specific parameters describing the demand, the capacity and observed free traffic speeds. In doing so, we define demand-to-supply ratio of a city as


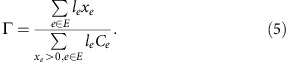


This dimensionless measure is a simple ratio of the total distance travelled by all vehicles to the upper bound of the total vehicle kilometres the road network can support per hour, thus capturing the load on the road infrastructure by bringing together trip distances, trip magnitudes, road capacities and the distances they span as shown in Table 1. Using this measure along with *v*_*f*_, the average free travel speed of each city, we are able to better explain the linear relationship between travel time and distance by


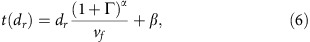


where *α*-values vary between 1.3 and 2.5, essentially describing the sensitivity of the city to the stress imposed by travel demand on its road infrastructure.

To untangle the particular ordering of cities in terms of speed and understand why some cities are more congested than others, we investigate a typical relationship in Fig. 3d, to test the common conception that cities with higher population densities tend to exhibit more heterogeneity in their demand profiles, and therefore tend to be more congested. For this purpose, we measure the ratio of the time lost in traffic to the travel time under free flow conditions, known as the traffic index, along with those measured for many other urban areas by TomTom, a leading GPS company. We consider the percentage of congestion, defined as the percentage of additional travel time due to traffic compared with free flow conditions, for different population densities in these various cities. We observe that Boston, Lisbon and Porto fall on the fit model, whereas the Bay Area and Rio demonstrates a significantly higher level of congestion. The outlier appearance of the Bay Area is a consequence of the arbitrary definitions of urban areas and its influence in population density as pointed out in ref. 4. To account for this, we plot the subdivisions of San Francisco and San Jose, which support the relationship, as they lie closer to the fit. Interestingly, the dimensionless demand-to-supply ratio Γ lacks this problem and presents a better linear trend with congestion for the five analysed urban areas as depicted in Fig. 3e, despite the broad behaviour of the traffic response. The two most congested cities have the highest ratios, the Bay Area closely followed by Rio de Janeiro, whereas Porto and Lisbon, the two least congested cities, have lower ratios.

To finalize our analysis, in Fig. 3f we measure how population densities are spatially distributed from the most densely populated region in each of the subject cities based on the chosen administrative level. The results show different spatial distributions in the population density of the five cities. First, it verifies the expected effect of higher population densities in increasing congestion. It also highlights the importance of the spatial distribution around the highest density point. Lisbon and Porto present densities of population below 500 people per km^2^ for distances of *r*>20 km, whereas the other three cities stabilize in values >1,000 people per km^2^. These differences can explain the two types of responses in the effective travel speeds presented in Fig. 3b, where Lisbon and Porto belong to a city type of lower density. Taking these results together, we observe that congestion increases with Γ and appears to be influenced by the spatial distribution of population density and its gradient.

### Selfish routing

In this section, we compare the travel times for commuters in free flow, socially optimal and user equilibrium flow configurations. Our findings in the five subject cities are outlined in Table 2. Although the estimated free travel time averages are similar, congestion plays a significant role: Lisbon commuters lose 2.1 min on average by selfish routing preferences. Rio de Janeiro exhibits an average loss of 2.6 min on average incurred by selfish routing. The results show that on average 15–30% of total minutes lost in congestion is caused solely by selfish routing.

**Figure 4:**
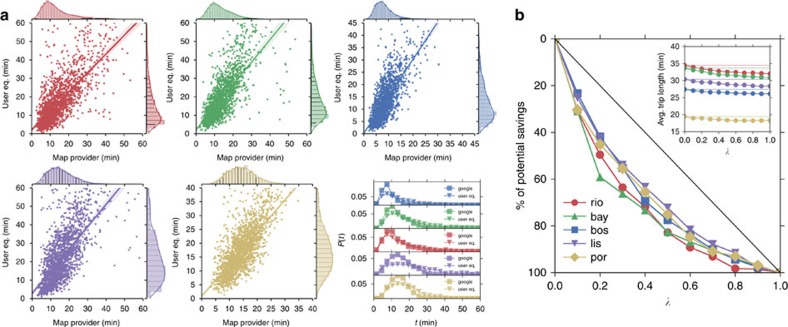
Travel time comparisons and potential savings. (**a**) Comparison of travel times and their distributions between user equilibrium versus routes obtained from the online map provider. OD samples consist of 2,000 OD pairs with the highest commuting flow magnitudes for each city. (**b**) The percentage of potential savings in average commuting times for the five cities for varying levels of social good weight of routing. (inset: the travel time savings represented in actual minutes).

Although a more nuanced methodology incorporating stochastic traffic assignment and probabilistic OD matrices would probably improve validation, our formulation and central findings would remain robust, as they are based on aggregate and endogenous, albeit simplified, behaviour of our system. Furthermore, a principled and singular validation source does not exist for our cities; we instead use an online map provider as a validation benchmark. Although the validation data are also the product of internal models and estimations, it is of value as they are obtained from an independent data source to ours. In Fig. 4a, we compare the distributions of obtained travel times with those obtained from the map provider in the morning peak hour between 7:30 and 8:30 h for 2,000 OD pairs with the highest commuting flows (see Supplementary Table 3 for statistics related to the regressions). There is an overall overestimation of travel times, which strengthens the notion that route choice in reality might not be a perfect user equilibrium or a social optimum, but somewhere in between. Neither the provider's nor our findings are expected to have accurate travel time variability, as these comparisons are estimates of typical travel times for the given OD pairs and they act as a first step towards the validation of our estimated travel times based on the assigned traffic flows obtained from the phone data.

### Weight of social good

In assessing the effects of socially aware routing behaviour for the subject cities, we calculate the average commuting time for various levels of *λ*. The inset of Fig. 4b depicts the decrease in average commuting travel times for increasing *λ* in all five cities, ranging from an average of 1–3 min. More importantly, the shape of the curves indicate that even modest social consideration weights can realize a significant portion of the potential savings. Figure 4b collapses these curves to represent realized potential savings as a percentage to exhibit a striking similarity between the five cities in terms of response to socially aware routing. To assess the economies of such routing behaviour, we measure the Gini index of the obtained curves; by definition, higher values of *G* indicate higher savings for smaller levels of social good weight. Our findings show that *G* ranges from 30–40%: *G*_rio_=41%, *G*_bay_=42%, *G*_bos_=33%, *G*_lis_=30% and *G*_por_=34%. These findings indicate congested cities benefit more from incorporating social good considerations into routing behaviour.

### Travel time benefit distributions

**Figure 5:**
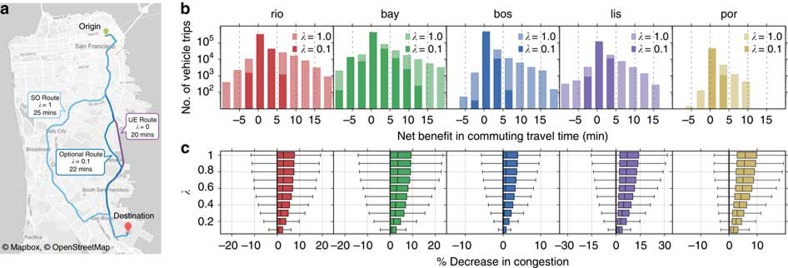
Benefit and congestion decrease distributions for different weights of social good. (**a**) A depiction of three route alternatives with the corresponding travel times for a trip from Union Square to San Francisco Airport for *λ*=0, *λ*=0.2 and *λ*=1, respectively. (**b**) Counts of vehicle trips and observed travel time benefits for *λ*=1 and *λ*=0.1. Negative benefits refer to increase in travel times for vehicles sacrificing for the social good. The spread of the distributions increase for higher *λ*. (**c**) The response of distributions of percentage decrease in time lost to congestion to increasing values of *λ*. The skewness towards positive values of congestion decrease indicate movement towards more optimal configurations. Maps under © OpenStreetMap contributors BY-SA.

In the previous section we characterized the percentage of potential savings that can be obtained for increasing levels of social consideration. However, these benefits are achieved at the expense of time of drivers who adjust their commute for the benefit of others. The unwillingness to give up time is the defining factor in drivers' failure to reach an optimal state on their own. This highlights the importance of fairness of the distribution of who has to sacrifice versus who benefits in terms of both the success potential of the implementation of policies or a reward/punishment reinforcement schema. Figure 5a demonstrates one such schema, where drivers are shown a route that corresponds to a choice, which might result in a travel time sacrifice.

Our findings, in accordance with the results of the previous sections, indicate a net bias towards benefits, meaning the number of drivers who benefit outnumber those who sacrifice. Figure 5b summarizes the benefit distributions for the five cities for *λ*=0.1 and *λ*=1. The former exhibits a less spread distribution than the latter but the skewness remains inherent to the distributions. Although the average benefits described in the previous sections appear small, it should be noted that 10-min benefits can be observed for tens of thousands of vehicles. Figure 5c describes in more detail how the positive skewness evolves for increasing social consideration. For higher *λ*, the % decrease in congestion distributions are shifted towards positive values, indicating a net benefit. This result demonstrates the potential of incentive schemes, which could compensate the few drivers who sacrifice under consideration of social good.

## Discussion

The economic and social costs of congestion are crippling. In addition to the overall loss of time, congestion underlies many major economic and urban issues such as increased gas consumption, infrastructure deterioration and CO_2_ emissions. In this work, we use massive amounts of data to estimate peak hour travel demand and understand travel times. We then explore the power of information-based routing on congestion alleviation.

Our findings suggest very interesting similarities in the behaviour of the five subject cities to explain congestion and potential benefits of social routing. Commuting distances follow a lognormal distribution and free travel speeds are normally distributed. A city's unique congestion fingerprint is strongly related to measurable characteristics. The population density and its spatial distribution together with the Γ parameter of demand-to-supply ratio are the two driving factors of the observed congestion in a diverse range of cities. Further, given the current state of traffic, we then estimate how centralized routing schemes using the power of information would reach possible benefits in travel times. Such information is important, as it allows the assessment of the upper bounds of routing policies; if effective in implementation, it would influence the traffic on a city scale. In practice, this would imply that we could have similar routing applications that we use today with the incorporation of demand profiles, to provide routes that are not necessarily the shortest but also the best for decreasing overall congestion.

We find that routing solutions that mimic socially optimal configurations, that is, *λ*=1, have a limit of decreasing time lost in congestion by up to 30%. This is in contrast with the effectiveness of direct and costly interventions where 1% target decrease in demand can achieve 18% decrease in travel times^18^. Although in both scenarios the collective benefits for the whole city can be significant (15–30% decrease), the observed time benefits the average individual receives are marginal, ranging from 1 to 3 min. Furthermore, these times are below the travel time variability based on events, weather conditions or traffic lights. Our findings indicate that in the best-case scenario, time savings would be imperceptible for the majority of the drivers. From this, it is clear that such routing solutions cannot fix the traffic problem for individual drivers but rather would contribute to the city as a whole. The advantage is that in the context of the implied routing application, the number of vehicles sacrificing their travel time is significantly smaller than the number of those that benefit. Lower levels of weight towards social good will also moderate the magnitude of benefits and losses, consequently making the policies fairer and easier to implement.

Open work in this subject contains, but is not limited to, a more generalized bottom-up approach to comparison of cities that includes various modes of transportation to demonstrate their similarities, differences and their consequences. As the volume, the variety and the resolution of data increase along with the expected disruptions from connected self-driving cars and similar technologies, this front of research will become more relevant to facilitate the study and planning for the future of urban mobility. With more updated demand models extracted from communication technologies, understanding the network effects on congestion will become easier to pinpoint and address. In addition, planning tasks on urban mobility previously difficult to tackle may now be addressed at lower costs and with much larger samples of the population. For example, a thorough analysis of how travel time and congestion is distributed among the population and its split by income and other sociodemographic characteristics remains an open front.

## Methods

### Mobile phone data

Mobile phone data sets, also referred to as CDRs, used in this study consist of at least 3 weeks of records of all mobile phone users of a particular carrier across each subject city. Each individual CDR consists of a hashed user identification string, a timestamp and the location of the activity. The spatial granularity of the data varies between cell tower level, where calls are mapped to tower locations and distributed uniformly within the Voronoi cell that it forms, and triangulated geographical coordinate pairs, where each call has a unique pair of coordinates accurate to within a few hundred metres. Market shares associated with the carriers that provide the data also vary (see Supplementary Figs 1 and 2, and Supplementary Note 1).

### Census and travel survey data

At the census tract (or equivalent) scale, we obtain the population, vehicle usage rate and median income of residents in that area. For US cities, the American Community Survey provides this data on the level of census tracts (each containing roughly 5,000 people). Census data are obtained for Brazil through IBGE (Instituto Brasileiro de Geografia e Estatística) and for Portugal through the Instituto de Nacional de Estatística. All cities analysed in this work have varying spatial resolutions of the census information. Wherever possible, we obtain the most recent travel demand model or survey from the subject city and compare the results with those output by our methods. We use the 2011 Massachusetts Household Travel Survey for Boston, 2,000 Bay Area Transportation Survey for the Bay Area and a recent transportation model output provided by the local government for Rio de Janeiro. For Lisbon, the most recent estimates from the MIT-Portugal UrbanSim LUT model that uses the 1994 Lisbon transportation survey as input are used. We found no recent travel survey or model for Porto (see Supplementary Note 2).

### Extraction of validated OD information

Traditional modelling approaches to OD information use data obtained from travel surveys, possibly combined with land-use and point-of-interest information, to generate estimates of trip production and attraction for locations. Although new data sources such as CDRs do not provide the same detailed demographic and contextual information about individuals or trips, they do provide many high-resolution data points over a far longer observation period. Mobile phones offer good, but imperfect measurements of geographic position due to the uncertainty of the location estimates and the non-uniform sampling frequency (see Supplementary Fig. 5 and Supplementary Note 3 for procedures to generate OD matrices and more descriptive information). For further questions and inquiries about the OD data, please contact the corresponding author.

### Road networks

For many cities in the United States, detailed road network data are made available by local or state transportation authorities. These data sets generally are well maintained; however, many properties are often incomplete or missing entirely. For this purpose, we infer required road characteristics to build realistic and routable networks using OpenStreetMap, an open-source crowd sourced mapping tool (see Supplementary Note 4).

### Traffic flow and travel time

Relating travel performance to traffic conditions has been a long-standing problem in transportation. Many different characterizations exist, ranging from conical volume-delay functions to more complex approaches (see Supplementary Fig. 4 and Supplementary Note 5).

### Traffic assignment

Traffic assignment is a mature domain that aims to bring together travel demand with road infrastructure, to better understand traffic, and has been studied extensively by urban and transportation planners. In this work, we follow an efficient, static, origin-based assignment algorithm that focuses on the equilibration of a directed acyclic graph structure emanating from every origin node (see Supplementary Fig. 6 and Supplementary Note 6).

## Additional information

**How to cite this article:** Çolak, S. *et al.* Understanding congested travel in urban areas. *Nat. Commun.* 7:10793 doi: 10.1038/ncomms10793 (2016).

## Supplementary Material

Supplementary Information Supplementary Figures 1-8, Supplementary Tables 1-3, Supplementary Notes 1-6 and Supplementary References.

## Figures and Tables

**Table 1:** A comparison of general properties of the subject cities.

	**City**				
	**Rio**	**SF Bay**	**Boston**	**Lisbon**	**Porto**
Population (millions)	12.6	7.15	4.5	2.8	1.7
Area (1,000 km^2^)	4.6	18.1	4.6	2.9	2.0
Demand (vehicle km h^−1^)	3.1	9.1	5.4	2.9	1.1
Supply (vehicle km h^−1^)	17.6	43.0	39.7	25.5	11.7
Demand-to-supply (Γ)	0.18	0.21	0.14	0.11	0.09
Expansion factor	890	100	32	96	164
Vehicle usage (vehicle per person)	0.25	0.67	0.67	0.56	0.62

**Table 2:** Comparison of cost findings in the subject cities for the morning peak hour.

	**City**				
(**min**)	**Rio**	**SF Bay**	**Boston**	**Lisbon**	**Porto**
FTT	20.6	21.1	19.3	22.4	15.3
**Loss**	**14.1**	**12.5**	**8.2**	**8.0**	**4.0**
UE	34.7	33.6	27.5	30.4	19.3
**Benefit**	**2.6**	**2.6**	**1.3**	**2.1**	**1.1**
SO	32.1	31.0	26.2	28.3	18.2
%S	18	21	16	27	28

FTT, free travel time; SO, social optimum; UE, user equilibrium; % S, percentage of total congestion attributed to selfish routing, defined as *S*=100*Benefit/Loss.

Bold rows indicate the loss of travel times from free travel times to socially optimal flows, and from socially optimal flows to user equilibrium flows for commuters, respectively.
