# Supplementary material for: Understanding congested travel in urban areas
Source: Nat Commun. 2016 Mar 15;7:10793. doi: 10.1038/ncomms10793 (PMC4796288; doi:10.1038/ncomms10793)
Supplement: Supplementary Information — Supplementary Figures 1-8, Supplementary Tables 1-3, Supplementary Notes 1-6 and Supplementary References. [file ncomms10793-s1.pdf]

ID	Time	Lat	Lon	Location
12345678	24/12/2012 09:00:00	42.397	-71.121	A
12345678	24/12/2012 10:00:00	42.360	-71.057	B
12345678	24/12/2012 10:05:00	42.360	-71.057	B
12345678	24/12/2012 12:00:00	42.360	-71.094	C
12345678	24/12/2012 16:00:00	42.360	-71.094	C
12345678	24/12/2012 20:00:00	42.397	-71.121	A
...	...	...	...	...

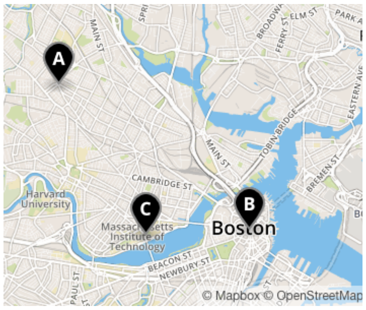

Supplementary Figure 1: A typical depiction of rows of CDR data in Boston. User 12345678 makes a call from location A (Davis square), then goes on to make two calls from location B (Boston City Hall), then makes one call location C (MIT) at noon and another later, and makes one final call again from the location A at 8pm.

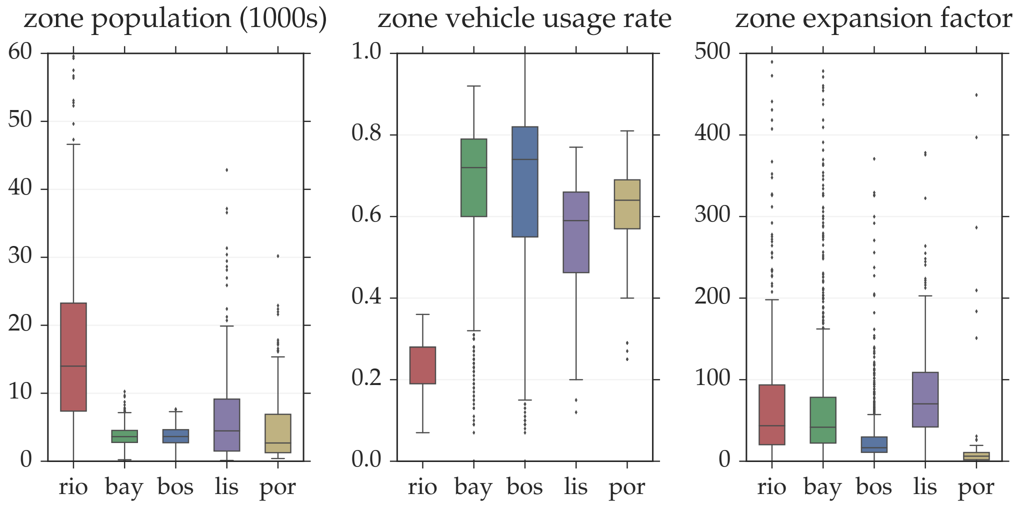

Supplementary Figure 2: The population, vehicle usage rate and the expansion factor distributions of the five subject cities.

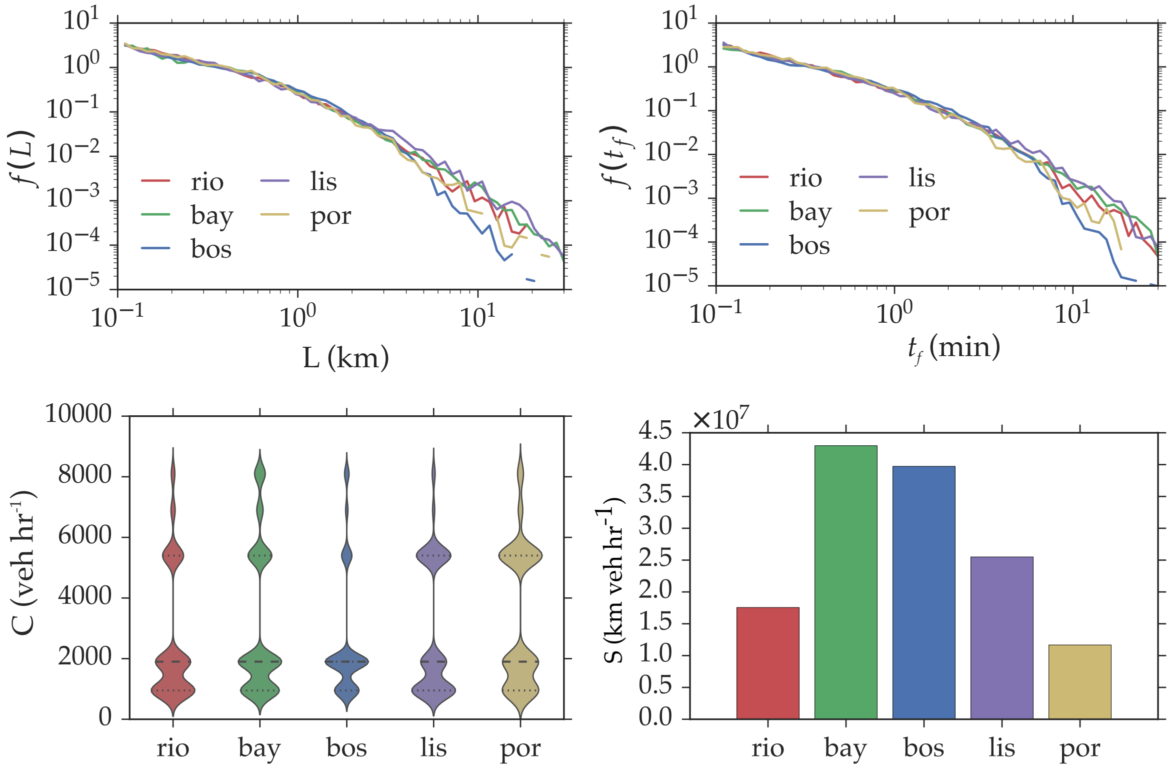

Supplementary Figure 3: Properties of the road networks of five subject cities. Distributions of road segment length L, free travel time t_f, hourly vehicle flow capacity C, and a measure of total supply S.

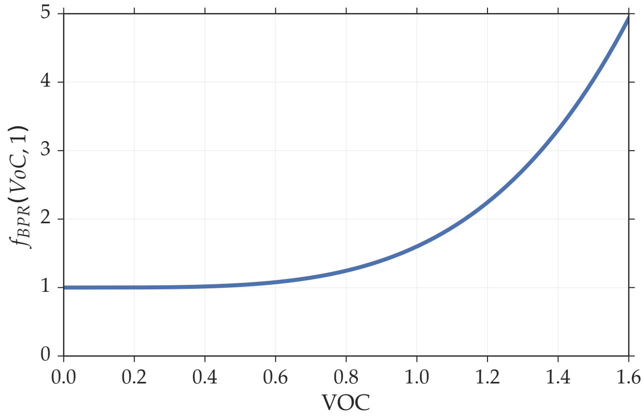

Supplementary Figure 4: BPR function for obtaining travel time from volume and capacity.

Algorithm B(N)

Initialize B as the shortest path tree rooted at the origin.
Assign all flows to links to B.
while $r_g > 0.001$
do {
  for all origins o
  do {
    Add to B_o edges e with negative reduced costs.
    Solve the Restricted Master Problem for B_o.
    Simplify B_o by removing $\{e|x_e = 0\}$.

Restricted Master Problem(Bush B, ϵ)

Update costs on all links on B.
Calculate the longest route tree with paths P_i and costs U_i.
Calculate the shortest route tree with paths p_i and costs u_i.
if $max\{U_i - u_i, \forall i\} \leq \epsilon$, stop.
else *continue*.
for all j
do {
  set of links in p_i not in P_i : $S_j = p_i \setminus P_i$
  set of links in P_i not in p_i : $L_j = P_i \setminus p_i$
  difference in costs to j : $g = (u_j - u_i) - (U_j - U_i)$
  total marginal cost of sets S_j and $L_j h = \sum_{e \in S_j \cup L_j} c_e^{'}$
  flow to be shifted : $dx = min\{g/h, min\{x_e|e \in L_j\}\}$
  add flow to shorter path : $x_e = x_e + dx, e \in S_j$
  remove flow to shorter path : $x_e = x_e - dx, e \in L_j$
  update travel times : $t_e, e \in S_j \cup L_j$

Supplementary Figure 5: Algorithms used in the computation of equilibrium. [1, 2]

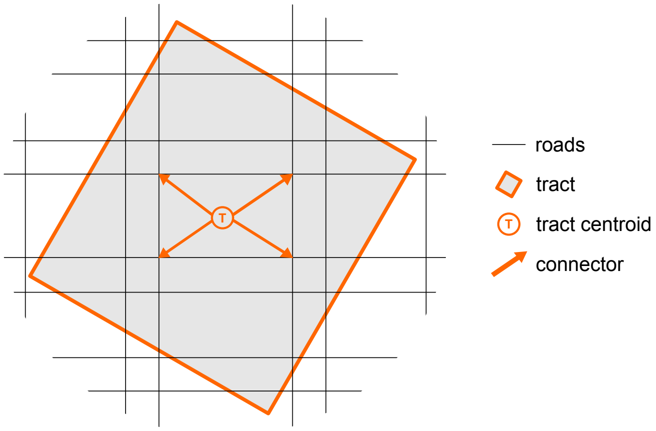

Supplementary Figure 6: A depiction of connector modeling. Tract centroid T is connected to nearest four intersections.

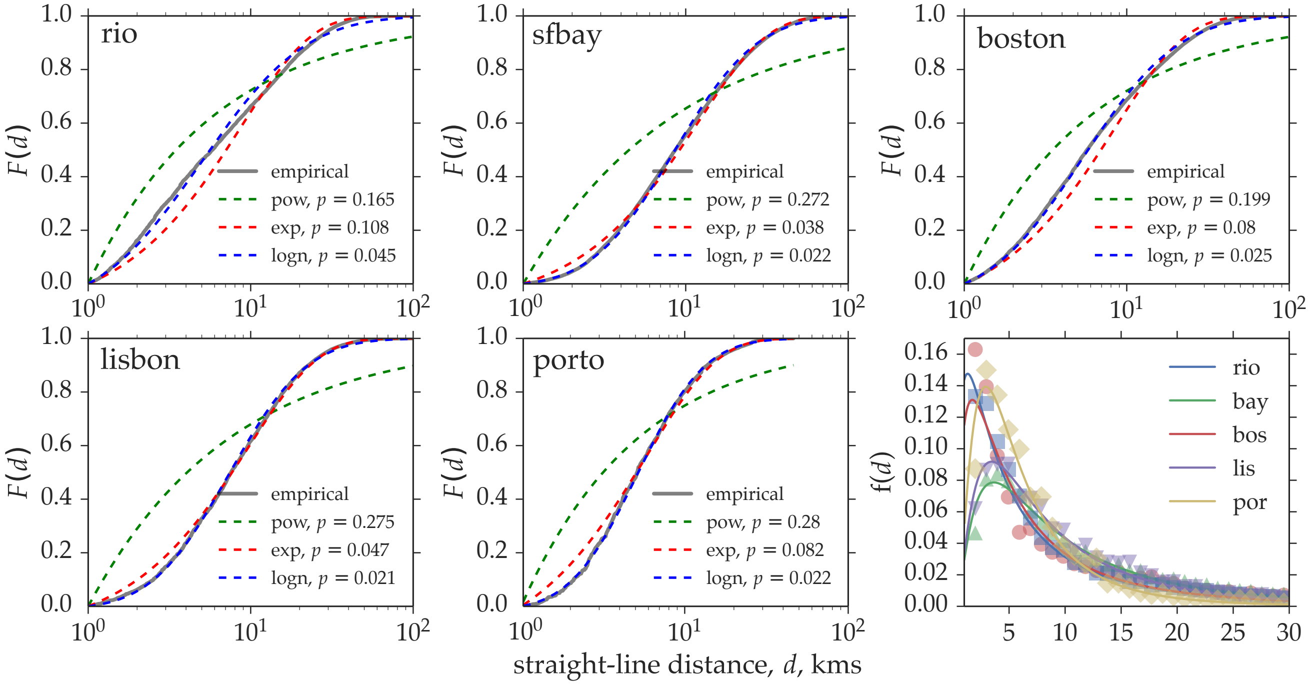

Supplementary Figure 7: The probability distribution fits for the straight-line distances.

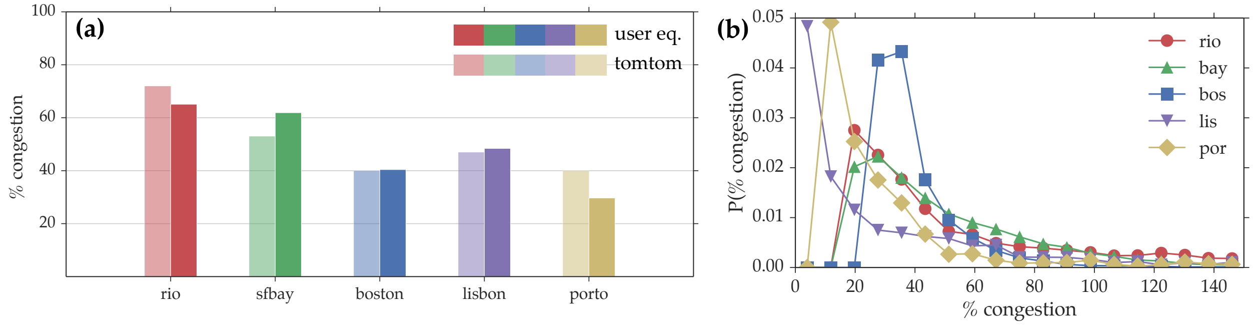

Supplementary Figure 8: % congestion distributions for the five cities. **(a)** Overall % congestion levels obtained fro user equilibrium compared to values from TomTom. **(b)** Distributions of % congestion for trips for the five cities.

	City				
	Rio	Bay	Bos	Lis	Por
population (mil.)	12.6	7.15	4.5	2.8	1.7
area (1000 km^2)	4.6	18.1	4.6	2.9	2.0
# of total users (mil.)	2.19	0.43	1.65	0.56	0.47
# of calls (mil.)	1045	429	905	50	33
data period	5 months	3 weeks	2 months	14 months	14 months
data type	tower	tower	lat-lon	tower	tower
# of cell towers	1421	892	N/A	743	335
# of edges (th.)	40.9	24.3	21.8	28.1	15.1
# of nodes (th.)	22.1	11.3	9.6	16.1	8.6
# of tracts	381	1139	732	295	272
roads (th.miles)	6	20	12	7	3
all trips (mil.)	0.432	1.015	0.916	0.324	0.171
commutes (mil.)	0.183	0.353	0.401	0.151	0.084

Supplementary Table 1: A comparison of the extent of the data involved in the analysis of the subject cities.

	straight-line distance fit, KS statistic				
	Rio	Bay	Bos	Lis	Por
power-law	0.138	0.193	0.142	0.200	0.177
exponential	0.087	0.035	0.082	0.016	0.026
lognormal	0.049	0.023	0.028	0.021	0.018

Supplementary Table 2: KS test statistics for lognormal, exponential and power-law distribution fits for the straight-line distances.

	travel time distributions, regression statistics				
	coef	st. error	$P > \mid t \mid$	R^2	AIC
Rio	0.7400	0.007	0.000	0.839	$1.239 * 10^4$
Bay	0.6490	0.006	0.000	0.876	$1.129 * 10^4$
Bos	0.5770	0.005	0.000	0.882	$0.904 * 10^4$
Lis	0.6297	0.006	0.000	0.854	$1.232 * 10^4$
Por	0.7602	0.005	0.000	0.922	$1.078 * 10^4$

Supplementary Table 3: Regression statistics for travel time estimations.

Supplementary Note 1

Mobile phone datasets, also referred to as CDRs (Call Detail Records), used in this study consist of the mobile phone activity logs of all mobile phone users across a specific carrier in every subject city. The nature of the activity varies: for all cities calls made by the user is included. Received calls, SMS activity, and various location signals may also be included. A minimum of three weeks of phone call records are available, although for some cities the period of the data is significantly longer. The granularity of the spatial component of the data in Rio de Janeiro is at the cell tower level: where calls are mapped to the Voronoi cells formed to model the coverage area of each tower. For other cities, the spatial information comes in triangulated latitude-longitude pairs, where each call has a unique pair of coordinates with and accuracy of roughly few hundred meters. Market shares associated with the carriers that provide the data also vary. Supplementary Table 1 compiles descriptive statistics for these data sources for each city we explore in this paper.

Each individual call detail record consists of a hash string identifying the mobile phone user, a timestamp marking the time of the activity, and the described spatial information regarding the activity. Supplementary Figure 1 depicts an example daily log of a user living in Boston, where the location field is inferred as unique locations visited.

CDR data inherently contains noise, as expected in any similar dataset. One reason for noise is the set of algorithms mobile phone carriers use for tower-to-tower call balancing to improve service. This operation creates discontinuities in the data that do not represent actual movement. To remove this noise and correct for other similar discrepancies, we apply a procedure generally used for GPS traces, referred to as a stay-point algorithm. Jiang et al. provide a thorough review of these techniques in [3] and we adapt the stay point algorithm originally described by Zheng et al. in [4]. In summary, stay-point algorithm simplifies a sequence of calls within a specified spatiotemporal area. In other words, calls within a certain radius and timeframe are bundled together. The *pass-by points* are removed, and *stays* remain. This mapping is made such that the representative point is the medoid of all such calls. For all cities here, except Boston where the data is triangulated, this algorithm is applied in a modified way. A tower-based CDR dataset only roughly describes the region from which the call was made, that is, the estimate of a user's position is only known up to the Voronoi cell for that tower. For this reason, the simplification of the series of calls is applied by serializing the calls made from towers within a certain distance. For the temporal dimension, these calls are labeled as stays only if the user is known to be in that location for at least 10 minutes.

One key point worth noting is that CDRs are of passive nature: except for a very tiny portion of the data, a mobile phone user's location information is only visible in the data when he/she interacts with his/her phone. Therefore it is certainly possible for a user to be in the location the data point classified as a pass-by, or

alternatively be visiting other locations that cannot be distinguished due to lack of phone interaction. This issue and other similar shortcomings resulting from the nature of the data are discussed in detail in previous work [5, 6, 7].

Supplementary Note 2

At the census tract (or equivalent) scale, we obtain the population and the vehicle usage rate of residents in that area. For US cities, the American Community Survey provides this data on the level of census tracts (each containing roughly 5000 people). Census data is obtained for Brazil through IBGE (Instituto Brasileiro de Geografia e Estatística) and for Portugal through the Instituto de Nacional de Estatística. All cities analyzed in this work have varying spatial resolutions of the census information.

Supplementary Figure 2 exhibits properties of the administrative boundaries used. Boston and Bay Area, regions in the United States, exhibit uniformity in their distributions of population per zone, as the populations are generally around 5000. Lisbon and Porto demonstrate higher deviations for a similar median, whereas the magnitude of the spread in Rio de Janeiro is higher than the other cities. To get an estimate of the vehicle usage rates, we use the following relationship:

$$VUR(i) = P_{\text{drive alone}}(i) + P_{carpool}(i)/S,$$

where $P_{\text{drive alone}}(i)$ and $P_{carpool}$ are probabilities that residents in zone i drive alone or share a car, respectively. $S = 2.5$ is estimated to be the average carpool size [8].

Conversely, Boston and Bay have the highest vehicle usage rates whereas in Rio de Janeiro people are less car-oriented. To assess how similar our five cities are in terms of CDR data sampling we compare their expansion factors, defined as the ratio of the number of people living in a tract to the number of people assigned that tract as a home location. All cities have a mean below 100, although outliers exist.

Supplementary Note 3

Origin-destination (OD) information is traditionally modeled with data obtained from travel surveys, land use information and census data. First, estimates of trip production and attraction for zones are produced. These trips are then distributed among possible destinations across the city using calibrated gravity or radiation or similar models. Information from the survey are combined with mode choice models to split trips among travel alternatives. CDRs do not provide as detailed demographic and contextual information about travel patterns and behavior as household travel surveys do. Mobile phones offer good, but imperfect measurements of geographic

position due to the uncertainty of the location estimates and the nonuniform sampling frequency. However millions of high resolution data points over a far longer observation period make CDRs a high potential data source. Methods developed to incorporate CDRs therefore aim to find a balance between a small and complete dataset that is household travel surveys, and a large but incomplete dataset, namely CDRs.

In incorporating CDRs into such methods, Alexander et al. and Colak et al. [6, 7], outline a general framework. Location frequencies are found to estimate each location's function for a user, and classify it as *home*, *work* or *other*. Consequently the trips between these locations are assigned a trip purpose: *home-based-work* (commuting, *home-based-other* or *non-home-based* are inferred. Morning peak commuting and total trips are estimated from filtered users by analyzing consecutive observations at different stay points during the morning peak period (6am-10am). These trips are then normalized to accurately represent actual daily number of trips by measuring how often a user uses their phone, their average number of trips, and the number of days that they were observed. Finally, the number of trips are expanded by the ratio of the population of the source tract to the number cell phone users in that tract. To consider trips made only by vehicles, we weigh obtained person trips by vehicle usage rates in the home census tract of users. To estimate the peak hour traffic volume, the morning period of to 6am-10am was weighted in accordance to trip departure time distributions obtained in [7]. Peak hour demand occurs between 7:30am and 8:30am, and the average morning hour demand is multiplied by 1.5 to reflect the peak as per the departure time distributions. Another issue relating to the accuracy of findings is the choice of the administrative boundaries, that is, due to the spatial precision of the data, certain aggregation levels work better than others. This problem is analyzed in detail in previous work, where pseudocode to generate OD matrices and the comparisons to the outputs of traditional models can also be found [6, 7, 5].

Supplementary Note 4

While road networks supplied by local municipalities in the form of shapefiles can often be useful, we have implemented a parser to construct routable road networks from OpenStreetMap (OSM) data due to its global availability. *Nodes* in OSM data represent points representing points of interest or tags or an intersection, and *ways* contain references to nodes that are grouped. They may also contain attributes such as *number of lanes* or *speed limit*, although many roads have this information missing. What all roads have in common though is the road classification, varying between motorway, trunk, primary, secondary, tertiary, residential and trunk roads, as well as a some other irrelevant categories. For our purposes, we filter out roads with irrelevant categories, and residential roads as they are not central to the congestion problem, yet tend to increase computation time significantly. For easing computation, we also simplify the network by collapsing roads with only one incoming and one outgoing road, if they're in the same road classification. To infer the missing data, we map assign every

road a speed limit, number of lanes and a corresponding capacity based on its category and information in [9]. *Motorways* are generally major highways and have a speed-limit of 60 mph with 3 lanes in a direction, whereas primary roads are 40mph with 2 lanes. We assume the free travel time on a segment i is $t_{f,i} = 1.3 * L_i/v_i$, with L_i the road segment length and v_i the speed limit. To estimate the capacity of a road segment, we utilize the following relationship [9] using the speed (kms/hr) and the number of lanes:

$$capacity,\ \ vehicles\ per\ hour = \begin{cases} 950\ veh/hr\ *\ \#\ of\ lanes, & \text{if } speed < 40\ mph, \\ (1500\ veh/hr + 30 \cdot speed) *\ \#\ of\ lanes, & \text{if } 40\ mph \leq speed < 60\ km/hr, \\ (1700\ veh/hr + 10 \cdot speed) *\ \#\ of\ lanes, & \text{if } speed \geq 60\ mph. \end{cases}$$

More information about the road networks can be found in Supplementary Table 1.

Road network modeling is a lot more complex then the simple extraction of the topology. Realistic estimation of road capacities, lengths and travel times is essential. We demonstrate our findings in Supplementary Figure 3. The road length and free travel times seem to follow a power-law, free travel times can range from ten seconds to as much as 20 minutes, and similarly for road lengths. Capacities are a direct result of road classes in OSM data: highways, trunks, primary, secondary and tertiary roads are all modeled to have different capacities and number of lanes. To assess overall supply more accurately, we also look at the product of the capacity and the length of the road networks. Our findings suggest that Bay Area, also in accordance with its size, has comparably larger supply.

Supplementary Note 5

A long-standing problem in highway engineering has been the characterization of the relationship between number of vehicles on a road segment, i.e. its *volume*, with the observed travel time on that road segment. Throughout the years a number of different characterizations have been developed ranging from conical volume-delay functions to more complex approaches [10, 11, 12]. One of the most simple and common metrics used in determining the travel time associated with a specific flow level is the ratio volume of vehicles on the road and its maximum flow capacity, also referred to as volume-over-capacity or VoC. At low VoCs, drivers enjoy large spaces between cars and can safely travel at free-flow speeds. As roads become congested and VoC increases, drivers are forced to slow down. Based on the guidelines set by the Bureau of Public Roads [13], the VoC of each road segment is used to estimate the travel time according to Eq. 1:

$$f_{BPR}(VoC, f_p) = t_f * \left(1 + \alpha\,(VoC)^{\beta}\right) * f_p, \tag{1}$$

where t_f refers to the travel time under free flow conditions. $\alpha = 0.6$ and $\beta = 4$ are calibration parameters. The relationship is depicted in Supplementary Figure 4. f_p is a city-specific correction factor: $f_p^{bos} = 1.4$, $f_p^{rio} = f_p^{bay} = 1.3$, and $f_p^{lis} = f_p^{por} = 1.0$.

As a second calibration step, once the path-level travel times are obtained, we adjust the travel times by

$$t_c = t + k_{city} * t_{free}, , \tag{2}$$

where $k_{bos} = k_{rio} = k_{bay} = -0.1$, $k_{lis} = 0$ and $k_{por} = 0.1$.

Supplementary Note 6

Traffic assignment is a very mature domain that has been studied extensively by urban and transportation planners. Static non-equilibrium models approaches consist of treating all users as homogeneous agents who make route choices prior to departure based on some heuristic related to current traffic conditions (e.g. the path that minimizes travel time). Incremental Traffic Assignment (ITA) is a variant of these static non-equilibrium assignment models that assigns batches of trips serially and updates costs between increments, as an improvement over the simplest all-or-nothing assignment methods. However, these methods results in solutions far from the Wardrop principles [14], where in the resulting system no driver should have an incentive to deviate from their route choice. Many methods to compute the equilibrium have been proposed in the literature [15], the easiest being from Frank-Wolfe (FW) solutions. FW based algorithms are quick to implement but slow to converge to the optimal solution. However they provide no information about which OD-pairs provide what amount of flow to which road segments. Path based algorithms take a step towards path enumeration, but in large networks with a high number of origin-destination pairs and alternative paths, the memory and computational requirement grow very quickly [16, 1, 17]. The more efficient approach is through the use of origin based algorithms, which are computationally feasible, have a fast convergence rate and do store path flows [18, 19]. More complex assignment models aim to take into account the variability in travel times by adding stochasticity to link travel times [20]. The process with which people choose routes is also of great interest to researchers, under the umbrella of route choice models. Prato (2009) presents a good overview of the wide literature on this subject, ranging from logit models to path set generation algorithms [21]. For the scope and the aggregate nature of our work, we opt to implement a static assignment model.

In this work, we will follow Algorithm B, proposed in [1] along with modifications and improvements outlined in [2], an origin based algorithm that focuses on the equilibriation of a graph structure referred to as a bush, a directed acyclic graph (DAG) emanating from every origin node introduced to the graph as the centroid of the origin tract. These structures are used with the reasonably assumption that in the equilibrium flows, no directed

cycles should exist as no driver has an incentive to increase his/her travel time. The computational efficiency of this algorithm stems from the fact that DAGs can be traversed in linear time. The algorithm used in this work is outlined in Supplementary Figure 5.

In these algorithms, the objective is to minimize the the distance between the current solution and the optimal solution. In this work, relative gap is used as the measure of convergence.

$$r_g = 1 - \frac{\sum_{o,d} t_{od} d_{od}}{\sum_{e \in E} t_e v_e}, \tag{3}$$

where t_{od} and d_{od} represent the demand and the travel time between an origin and a destination, and t_e and v_e represent the travel time and the volume on a road segment e. The numerator and the denominator essentially measure the same thing: the total travel time in the system. Theoretically, r_g is supposed to be equal to zero. This ensures that all drivers in the system are in fact taking the shortest possible routes, and the optimization problem is fully solved. Traffic assignment algorithms aim to bring r_g as close to zero as possible.

A critical design element of the implementation of origin based algorithms is the modeling of tract centroids, representing an aggregation of all the actual origins and destinations within the area, and the connectors, the hypothetical segments representing driver movement within the tract before joining the modeled road network [22]. Supplementary Figure 6 depicts the implementation of connectors in this work, where tract centroids are connected to the four nearest intersections.

Supplementary References

[1] Robert B Dial. A path-based user-equilibrium traffic assignment algorithm that obviates path storage and enumeration. *Transportation Research Part B: Methodological*, 40(10):917–936, 2006.

[2] Yu Marco Nie. A class of bush-based algorithms for the traffic assignment problem. *Transportation Research Part B: Methodological*, 44(1):73–89, 2010.

[3] Shan Jiang, Gaston A Fiore, Yingxiang Yang, Joseph Ferreira Jr, Emilio Frazzoli, and Marta C González. A review of urban computing for mobile phone traces: current methods, challenges and opportunities. In *Proceedings of the 2nd ACM SIGKDD International Workshop on Urban Computing*, page 2. ACM, 2013.

[4] Yu Zheng and Xing Xie. Learning travel recommendations from user-generated gps traces. *ACM Transactions on Intelligent Systems and Technology (TIST)*, 2(1):2, 2011.

[5] Jameson L. Toole, Serdar Colak, Bradley Sturt, Lauren P. Alexander, Alexandre Evsukoff, and Marta C. Gonzlez. The path most traveled: Travel demand estimation using big data resources. *Transportation Research Part C: Emerging Technologies*, 58:162 – 177, 2015.

[6] Lauren Alexander, Shan Jiang, Mikel Murga, and Marta C. Gonzlez. Origindestination trips by purpose and time of day inferred from mobile phone data. *Transportation Research Part C: Emerging Technologies*, 58, Part B:240 – 250, 2015. Big Data in Transportation and Traffic Engineering.

[7] Serdar Çolak, Lauren P. Alexander, Bernardo G. Alvim, Shomik R. Mehndiretta, and Marta C. González. Analyzing cell phone location data for urban travel: Current methods, limitations, and opportunities. *Transportation Research Record: Journal of the Transportation Research Board,*. in press.

[8] Pu Wang, Timothy Hunter, Alexandre M Bayen, Katja Schechtner, and Marta C González. Understanding road usage patterns in urban areas. *Scientific reports*, 2:1001, 2012.

[9] Highway capacity manual 2010. Technical report, Washington, D.C.: Transportation Research Board, 2010.

[10] David Branston. Link capacity functions: A review. *Transportation Research*, 10(4):223–236, 1976.

[11] Heinz Spiess. Technical note conical volume-delay functions. *Transportation Science*, 24(2):153–158, 1990.

[12] Rahmi Akcelik. Travel time functions for transport planning purposes: Davidson's function, its time dependent form and alternative travel time function. *Australian Road Research*, 21(3), 1991.

[13] Traffic Assignment Manual. Bureau of public roads. *US Department of Commerce*, 1964.

[14] John G. Wardrop. Some theoretical aspects of road traffic research. In *Proc. Inst. Civ. Eng.*, volume 1, pages 325–378, Part 2, 1952.

[15] P. Patriksson. *The Traffic Assignment Problem: models and methods*. V.S.P. Intl Science, 1994.

[16] R Jayakrishnan, Wei T Tsai, Joseph N Prashker, and Subodh Rajadhyaksha. A faster path-based algorithm for traffic assignment. *University of California Transportation Center*, 1994.

[17] Bruce N Janson. Dynamic traffic assignment for urban road networks. *Transportation Research Part B: Methodological*, 25(2):143–161, 1991.

[18] Larry J LeBlanc, Edward K Morlok, and William P Pierskalla. An efficient approach to solving the road network equilibrium traffic assignment problem. *Transportation Research*, 9(5):309–318, 1975.

[19] Masao Fukushima. A modified frank-wolfe algorithm for solving the traffic assignment problem. *Transportation Research Part B: Methodological*, 18(2):169–177, 1984.

[20] Carlos F Daganzo and Yosef Sheffi. On stochastic models of traffic assignment. *Transportation science*, 11(3):253–274, 1977.

[21] Carlo Giacomo Prato. Route choice modeling: past, present and future research directions. *Journal of Choice Modelling*, 2(1):65–100, 2009.

[22] Zhen Sean Qian and HM Zhang. On centroid connectors in static traffic assignment: Their effects on flow patterns and how to optimize their selections. *Transportation Research Part B: Methodological*, 46(10):1489–1503, 2012.
